# Plant- and Animal-Derived Organic Waste as Fillers in Biodegradable Composites for Advanced Applications: A Comprehensive Overview

**DOI:** 10.3390/polym18010022

**Published:** 2025-12-22

**Authors:** Roberto Scaffaro, Francesco Paolo La Mantia, Giada Lo Re, Vincenzo Titone, Maria Clara Citarrella

**Affiliations:** 1 Department of Engineering, University of Palermo, Viale delle Scienze, ed. 6, 90128 Palermo, Italy; vincenzo.titone@unipa.it (V.T.); mariaclara.citarrella@unipa.it (M.C.C.); 2 INSTM, National Interuniversity Consortium for Materials Science and Technology, Via Giusti 9, 50125 Florence, Italy; 3 Department of Industrial and Materials Science, Chalmers University of Technology, Rännvägen 2A, 41258 Gothenburg, Sweden; giadal@chalmers.se; 4 Wallenberg Wood Science Centre, Chalmers University of Technology, Kemigården 4, 41258 Gothenburg, Sweden; 5 Department of Civil and Industrial Engineering, University of Pisa, L.go Lucio Lazzarino 2, 56126 Pisa, Italy

**Keywords:** biodegradable biocomposites, circular economy, green composites, natural fillers, sustainable materials, waste valorization

## Abstract

Biodegradable polymeric composites reinforced with natural fillers represent one of the most promising routes toward low-impact, circular, and resource-efficient materials. In recent years, a growing number of studies have focused on the valorization of plant- and animal-derived organic waste, ranging from agricultural residues and natural fibers to marine and livestock by-products. This review provides a comprehensive and comparative overview of these systems, analyzing the nature and origin of the waste-derived fillers, their pretreatments, processing strategies, and the resulting effects on mechanical, thermal, functional, and biodegradation properties. Particular attention is dedicated to the role of filler composition, morphology, and surface chemistry in governing interfacial adhesion and end-use performance across different polymeric matrices, including PLA, PCL, PBS, PHA, PHB, PBAT, and commercial blends such as Mater-Bi^®^. The emerging applications of these biocomposites, such as packaging, additive manufacturing, agriculture, biomedical uses, and environmental remediation, are critically discussed. Overall, this work provides fundamental insights to support the development of the next generation of biodegradable materials, enabling the sustainable valorization of organic waste within a circular-economy perspective.

## 1. Introduction

**Figure 1:**
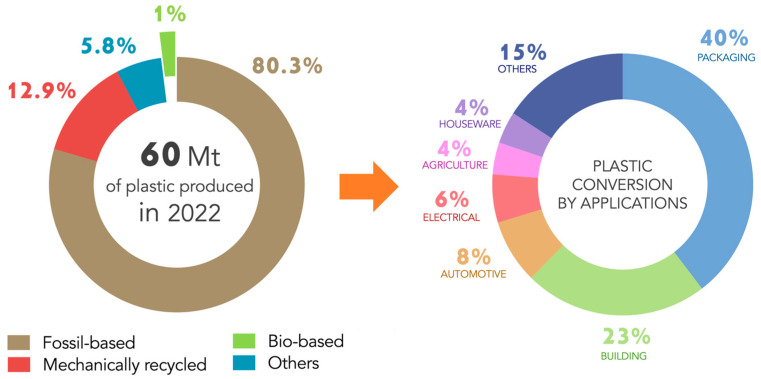
European plastic production in 2022 and its conversion into plastic products by applications. Data from 2022 Plastics Europe estimations—Eurostat [4].

The global dependence on petroleum-derived plastics has led to unprecedented levels of environmental pollution, resource depletion, and waste accumulation [1,2]. Over the past decades, plastic production has grown exponentially, surpassing 400 million tons per year, while recycling rates have remained critically low [3]. Figure 1 provides an overview of European plastic production in 2022 (60 Mt) and its subsequent conversion into different application sectors. As shown, the European plastics sector alone remains strongly dominated by fossil-based and non-biodegradable polymers (80.3%), which continue to accumulate in landfills and natural ecosystems due to their slow degradation rates and inefficient end-of-life management strategies. Only a minor fraction derives from bio-based feedstocks (1%) or mechanically recycled materials (12.9%). The right-hand chart highlights how polymeric materials are predominantly used in packaging (40%), followed by the building sector (23%) and a range of industrial applications including automotive, electrical, agriculture, and household products [4]. Overall, the figure underscores the continued reliance on fossil-based polymers. In parallel, increasing awareness and stricter environmental regulations are intensifying the demand for sustainable alternatives that can reduce the ecological footprint of materials while maintaining performance standards.

Within this context, biodegradable and bio-based polymers have emerged as promising candidates to replace traditional plastics in a wide range of applications. Polymers such as polylactic acid (PLA), polycaprolactone (PCL), poly(butylene succinate) (PBS), polyhydroxyalkanoates (PHAs), polyhydroxybutyrate (PHB), poly(butylene adipate-co-terephthalate) (PBAT), and thermoplastic starch combine a reduced environmental impact with mechanical and thermal properties often comparable to those of conventional petrochemical polymers [5,6,7]. However, despite these advantages, biopolymers still present important limitations, including brittleness, limited thermal resistance, moderate barrier properties, and relatively high production costs, which can restrict their technological applicability in demanding sectors [5,6,7]. To overcome these limitations, the scientific community has increasingly focused on biocomposites, materials obtained by reinforcing biodegradable polymer matrices with natural fillers [8]. This approach enables the development of systems that are not only more sustainable but also exhibit enhanced mechanical, thermal, functional, and degradation properties. More in detail, the addition of low-cost fillers improves stiffness, strength, or toughness [9,10,11], may enhance barrier or antioxidant capabilities, enable environmental remediation ability [12], and can modulate biodegradation kinetics [13,14]. Importantly, the environmental benefits are maximized when the fillers themselves originate from waste biomasses, enabling a dual advantage: valorizing organic waste and lowering the environmental and economic cost of composite production.

In this framework, plant- and animal-derived organic waste represents an abundant, renewable, and largely underutilized resource. Natural fillers include different types of materials: from agricultural residues such as peels, husks, fibers, and lignocellulosic fractions, to marine and animal by-products, including shells, bones, gelatin, and wool. These materials are generated in massive quantities globally. Agro-food industries produce millions of tons of lignocellulosic residues annually, while fisheries, aquaculture, and livestock sectors generate shells, bones, feathers, and wool scraps that are often disposed of at high environmental and economic cost [15,16]. When properly treated, these waste-derived fillers offer mechanical reinforcement, functional properties, or may act as nucleating agents that accelerate polymer crystallization and biodegradation [17]. Plant-derived wastes, rich in cellulose, hemicellulose, and lignin, generally show good compatibility with biodegradable polyesters and are valued for their reinforcing effect, low density, and availability at negligible cost [18]. On the other hand, animal-derived wastes contain minerals (e.g., CaCO_3_ in shells, hydroxyapatite in fish bones) and proteins (e.g., collagen, keratin, chitin), which can impart unique properties such as increased stiffness, bioactivity, biocompatibility, or enhanced degradability [15]. Furthermore, several studies have demonstrated that these fillers can enable advanced functionalities, making biocomposites suitable for packaging, agriculture, biomedical devices, additive manufacturing (3D printing), air filtration, and environmental remediation [18,19].

Despite the growing interest and the remarkable progress achieved in the field, several challenges remain unresolved. Organic waste streams are inherently heterogeneous in chemical composition, particle size, moisture content, and morphology. These factors strongly influence their compatibility with polymer matrices and the reproducibility of the final composites. Pretreatments are not always adopted, leading to difficulties in comparing results across studies. Additionally, the correlation between filler characteristics, processing methods, and final composite properties is not yet fully understood and often requires systematic investigation. Finally, while biodegradation is a key feature of these systems, comprehensive environmental assessments, such as life-cycle analysis (LCA), are still underrepresented in the literature.

The valorization of organic waste within polymer composites aligns with global strategies for the circular economy, zero-waste approaches, and resource-efficient materials design. By transforming residues into functional components, waste is reconceptualized not as a disposal issue but as a valuable raw material contributing to high-performance bio-based materials. This approach not only minimizes environmental impacts but also offers economic benefits for industries seeking sustainable alternatives and novel value chains.

Based on these considerations, this review aims to provide a comprehensive overview of the use of plant- and animal-derived organic waste as fillers in biodegradable biocomposites. Specifically, it analyzes the following:

i. The types, characteristics, and pretreatments of natural fillers;
ii. The processing strategies employed across different polymer matrices;
iii. The resulting mechanical, thermal, morphological, functional, and degradation properties;
iv. The main applications and end-of-life scenarios;
v. Current challenges, limitations, and future research directions.

By mapping the state of the art across plant and animal fillers, this review aims to offer a solid basis for the design of next-generation biocomposites and to stimulate further research toward the sustainable valorization of organic waste resources.

## 2. Biodegradable Polymeric Matrices for Biocomposites Production

**Figure 2:**
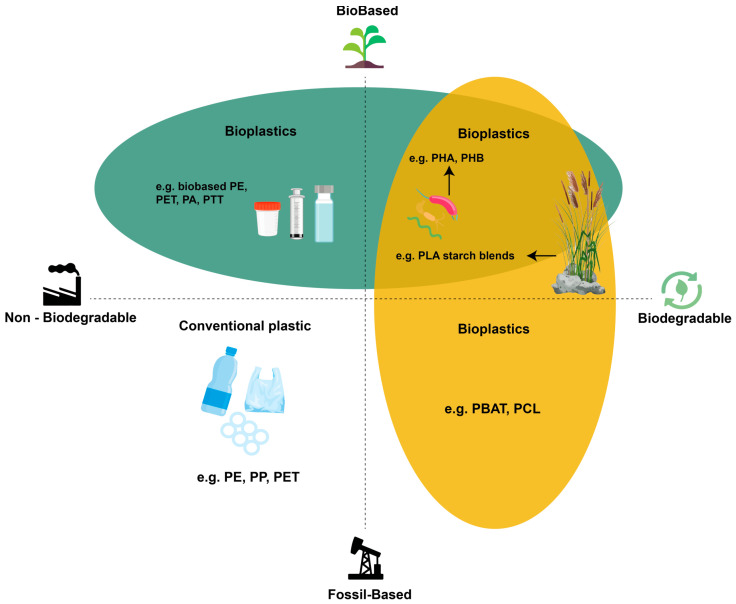
Overview of the classification of polymeric materials according to their origin (bio-based vs. fossil-based) and end-of-life behavior (biodegradable vs. non-biodegradable).

Polymer composites can fall into different categories depending on both the origin of the matrix and their end-of-life behavior, as illustrated in Figure 2. Materials may be bio-based or fossil-based, and independently, they may be biodegradable or non-biodegradable. This classification highlights that “bioplastics” is an umbrella term that includes multiple families of materials: bio-based but non-biodegradable composites (such as those derived from bio-PE or bio-PET), fully bio-based and biodegradable systems (e.g., PLA, starch-based blends, PHAs), and even fossil-based biodegradable polymers such as PBAT and PCL. Understanding these distinctions is crucial when designing sustainable biocomposites, since the environmental profile of a material depends not only on the renewable origin of its constituents but also on its degradability and disposal routes.

Biodegradable polymers are a class of materials considered a promising alternative to conventional polymers. Indeed, their main advantage is their ability to biodegrade under suitable conditions, which helps reduce plastic waste and environmental impact. Moreover, many of these materials offer performance that is comparable to, or sometimes even better than, conventional polymers. The most commonly available on the market and reported biodegradable polymers include polylactic acid (PLA) [20,21,22,23,24,25,26], poly-caprolactone (PCL) [27,28,29], poly(butylene succinate) (PBS) [30,31,32,33], polyhydroxyalkanoates (PHAs) [34,35], poly(butylene adipate-co-terephthalate (PBAT) [36,37,38,39], and thermoplastic starch (TPS) [40,41]. Each polymer presents different characteristics and can be used in various industrial fields. Undoubtedly, PLA is one of the most widely used polymers, partly due to its widespread availability on the market. However, PLA, produced through the fermentation of sugars, is also valued for its stiffness, transparency, and good processability, and is widely used in packaging, disposable products, textiles, and biomedical items, although it still has limitations for more advanced engineering applications [20,21].

Equally used mainly in biomedical applications, controlled release systems, and as a component in polymer blends to improve toughness and processability, is PCL [27,29], a biodegradable polyester with a low melting point and excellent flexibility. Instead, PBS produced from renewable or fossil-based monomers combines biodegradability with good thermal and mechanical performance and is used in films, containers, and coatings [30,31]. PHAs form a large family of polyesters produced by microorganisms using renewable carbon sources. Their properties can differ widely depending on the specific type; for example, poly(3-hydroxybutyrate) (PHB) is typically tough and brittle [35], whereas copolymers such as poly(3-hydroxybutyrate-co-3-hydroxyvalerate) (PHBV) offer improved flexibility and toughness [42].

As regards the biodegradable polymers obtained from fossil monomers, PBAT is a biodegradable aromatic–aliphatic copolyester used in the production of films, shopping bags, agricultural films, and compostable packaging [37]. It is characterized by high flexibility, strength, and good compatibility with other biodegradable polymers, particularly PLA and TPS [43]. The latter, obtained from starch sources such as corn or potatoes, is flexible, biodegradable, and particularly suitable for food packaging, edible films, and simple consumer products [40,41].

## 3. Natural Waste Fillers in Biocomposites

**Figure 3:**
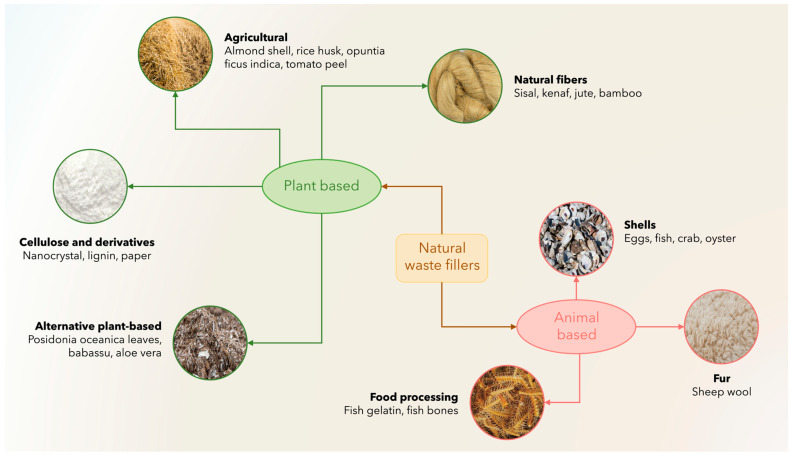
Classification of natural waste fillers used in biocomposites, grouped into plant-based and animal-based categories.

The development of biocomposites involves the use of natural raw materials and processing methods to improve their performance and sustainability. This section introduces the main categories of organic waste, both plant and animal, used as fillers, together with the processing strategies adopted to date to improve their compatibility and functionality. Figure 3 provides an overview of the wide variety of natural waste fillers currently explored for biocomposite production.

These materials can be grouped into plant-based and animal-based resources, each encompassing several derived subcategories. Plant-based fillers include agricultural residues (such as almond shells, rice husk, and tomato peels) [16,18], natural fibers (e.g., sisal, kenaf, jute, bamboo) [44,45], cellulose-rich derivatives (lignin, paper waste, nanocellulose) [46,47], and alternative plant by-products such as *Posidonia oceanica* leaves or babassu fibers [48,49]. On the other hand, animal-based sources include shells from poultry and seafood processing (eggs, crab, oyster) [50], fish waste (fish gelatin, bones), and wool or fur residues from livestock or the textile sector [8,15]. This classification highlights the wide diversity and abundance of natural waste streams available for sustainable composite manufacturing, emphasizing how both plant- and animal-derived by-products can be effectively valorized as functional fillers.

### 3.1. Plant-Derived Organic Waste

Plant-derived organic waste includes a wide variety of plant residues, which can be classified into four main groups: agricultural and agrifood waste, natural fibers, cellulose and derivatives, and alternative organic waste.

#### 3.1.1. Agricultural and Agrifood Waste

Currently, the valorization of agricultural and agrifood waste is a key strategy to promote the sustainability of their supply chains. Indeed, it is estimated that around 180 million tons of agricultural and agrifood waste are generated each year in Europe [16], of which a significant part is made up of residues, such as peels, stems, seeds, skins, and shells, from collecting and processing. However, the most recent data estimate that approximately 40% of them are currently not properly valorized [16]. As a result, the possibility of valorizing this waste is increasingly a real opportunity. Nonetheless, they range from fibrous materials to more compact and denser fractions with irregular particle size and variable porosity. For this reason, to make them compatible with processing, it is necessary to subject them to preliminary treatments, such as drying, grinding—to powder or particles—and, in some cases, even chemical treatments or addition of additives and/or compatibilizers to improve adhesion to the matrices. In Table 1, relevant studies regarding biocomposites based on agricultural waste fillers are reported.

Almost 61.7 % of the agricultural and agrifood waste used originates from Europe, 27.7 % from Asia, 8.5 % from South America, and 2.1 % from North America. Among the agricultural by-products most employed to produce biodegradable composites, rice husk [64,65,66,67,68,82,83] and nutshell-derived fillers [56,57,78,80,83,88,89,91,95] such as hazelnut and almond shells are among the most widely investigated. Fruit-processing residues, especially peels and seeds, are also widely explored as bio-fillers, with a particular emphasis on citrus by-products and other fruit species that are characteristic of specific local supply chains [52,55,59,63,70,74,76,81,86,90]. Recent studies also report fillers obtained from crop harvesting residues, such as tomato [71,94], corn [85,87], artichoke [51], and other vegetable by-products [69,79], as well as wastes generated during olive processing for table olives and oil production [60,74]. Several studies have also explored natural fillers derived from Mediterranean plant species, including *Opuntia ficus indica* [61,62,93] and *Hedysarum coronarium* [58,92], which have gained attention due to their abundance, low cost, and easy incorporation in biodegradable polymer matrices. Additional examples include fillers obtained from cocoa-processing residues [53,54,77], such as bean shells and husks. Moreover, several other isolated cases of agricultural waste filler have been reported in the literature [72,73,75,83,84], which highlight the broad diversity of agrowaste-derived materials being investigated for biodegradable composite formulations.

**Figure 4:**
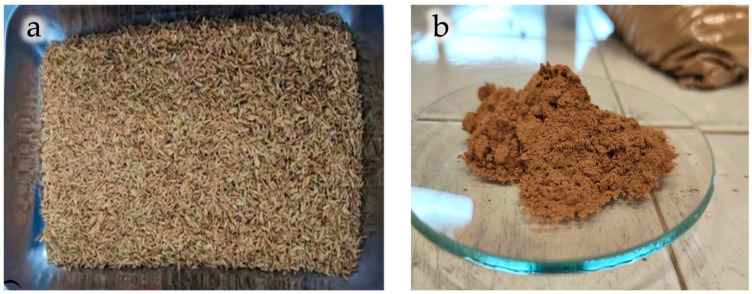
Ground rice husks (**a**) and hazelnut shells powder (**b**). Reprinted (adapted) with permission from [65,91].

The mechanical pretreatments used to prepare the filler involved grinding or knife milling, sometimes also ultracentrifugal (UC) [56,60,70,83] or high speed (HS) [62,95] milling, followed by sieving. Drying was typically carried out at temperatures ranging from 40 °C up to 80 °C, with some studies reaching temperatures above 100 °C [66,82,88,89] with drying times between 6 h and 72 h. In some cases, the drying step was not specified or was omitted. Some examples of ground waste materials are shown in Figure 4.

Moreover, in certain studies, additional treatments, such as alkali treatment or coupling agent application, were also performed. For instance, rice husk (RH) has been treated with a coupling agent to improve the properties of the biocomposites [64]. Similarly, in another study [65], both the rice husk (RH, Figure 4a) and PLA were treated with silane coupling agents (KH550 and KH570) to enhance the interfacial surface quality. Even for rice straw [66,67], tangerines [70], apple pomace [76], and almond shells [78,80], coupling agents were applied to improve interfacial adhesion. Alkaline treatments were also performed to remove undesired components from tomato peels (THP) and pineapple leaf (PL) [71,81].

#### 3.1.2. Natural Fibers

**Figure 5:**
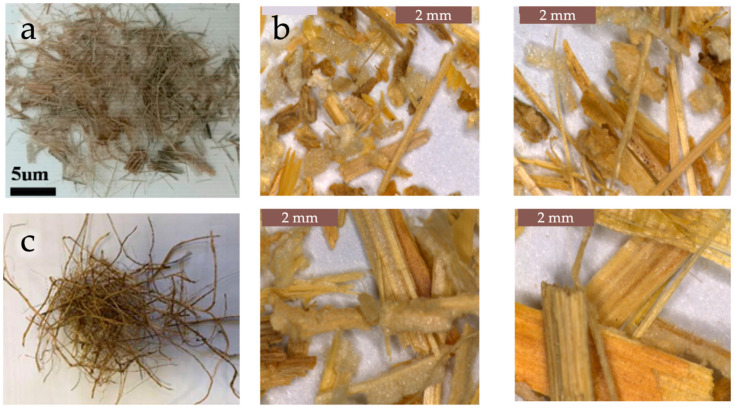
Close-up photos of abutilon indicum (**a**), corn stalk (**b**), and date palm (**c**) fibers. Reprinted (adapted) with permission from [96,100,118].

A valid alternative to synthetic fibers is today represented by natural fibers, which, thanks to their biodegradability and especially their low cost, are increasingly used in various applications (see Figure 5). Table 2 offers a comprehensive summary of studies focused on the use of natural fibers for biodegradable composites production. Only 28.2% of the works consider fibers originating from Europe, while 48.7% is from Asia, 15.4% is from South America, and 7.7% is from Africa.

**Table 2:** Matrices, fillers, and treatments of biocomposites based on natural fiber waste.

Matrix	Filler	Sample Code	Area	Type (*)	Diameter [µm]	Length [mm]	Density [g/cm^3^]	Other Treatments	Additive	Ref.
PLA	Abutilon indicum	PLA/AI	Asia	D	-	2.5	-	-	-	[96]
PLA	Agave	PLA/AF	South America	-	-	-	-	-	-	[97]
PLA	Bamboo	PLA/BF	Asia	D	-	2–6	-	Chemical	-	[98]
PLA	Bamboo	PLA/BF	Asia	D	-	2–6	-	Chemical	-	[99]
PLA	Corn stalk	PLA/CS	Europe	D	-	1–4	-	-	-	[100]
PLA	Elephant grass	PLA/EG	Asia	D	250	3	-	Mercerization and bleaching	-	[101]
PLA	Flax	PLA/CFY	Asia	C	400	20	-	-	-	[102]
PLA	Flax	PLA/Flax	Europe	D	-	-	-	-	-	[103]
PLA	Flax	PLA/FS	Europe	C	-	-	1.47	-	-	[104]
PLA	Flax	PLA/FS	Europe	D	300–600	2–5	-	-	Plasticizer	[105]
PLA	Hemp shives	PLA/HS	Europe	D	-	<1	1.51	-	-	[106]
PLA	Himalaya calamus falconeri	PLA/THF	Asia	D	-	3–5	-	Mechanical extraction	-	[107]
PLA	Jute	PLA/Jute	Europe	D	-	-	-	-	-	[103]
PLA	Kenaf	PLA/KF	Asia	D	250	-	-	-	-	[108]
PLA	Kenaf	PLA/KF	Asia	D	70–250	-	-	-	-	[109]
PLA	Kenaf	PLA/KFA	Asia	D	-	-	-	Acetylation	Acetic anhydride	[110]
PLA	Kenaf	PLA/LK	Asia	C	-	175	-	-	-	[111]
PLA	Kenaf (woven)	PLA/WK	Asia	D	-	-	-	-	-	[112]
PLA	Pennisetum setaceum	PLA/PS	Europe	D	75	1–2	-	-	-	[113]
PLA	Sisal	PLA/SF	Asia	D	-	3–6	1.24	Pretreatment	-	[114]
PLA	Sisal	PLA/SF	Asia	D	-	3–8	-	-	-	[115]
PLA	Sisal	PLA/SF	Africa	D	239	-	-	-	-	[116]
PLA	Sisal	PLA/MS	Africa	D	239	-	1.42	-	-	[117]
PCL	Date palm	PCL/DP	Asia	D	-	10	0.9–1.2	-	-	[118]
PCL	Hemp	PCL/HF	Europe	D	22	<1	-	-	-	[119]
PCL	*Phoenix* *dactylifera* L.	PCL/DP	Asia	D	-	10	0.92	-	-	[120]
PBS	Curaua	PBS/C	South America	D	-	10–40	-	-	-	[121]
PBS	Hemp	PBS/HF	Europe	D	-	30	-	-	-	[122]
PHA	Pineapple leaf	PHA/PLF	Asia	D	300–450	-	-	-	-	[81]
PHB	Sisal	PHB/SF	Asia	D	-	-	-	-	-	[123]
PHBV	Alfa	PHBV/AF	Africa	D	-	-	-	Chemical	-	[124]
PBAT	Croton lanjouwensis	PBAT/CF	South America	D	-	-	1.5	-	-	[125]
PBAT	Malvastrum tomentosum	PBAT/MF	South America	D	-	-	1.5	-	-	[125]
PBAT	Trema micrantha	PBAT/TF	South America	D	-	-	1.5	-	-	[125]
PBAT	Cannabis sativa	PBAT/CS	Europe	D	<32	-	-	-	-	[126]
PBAT	Hemp	PBAT/HF	Asia	D	-	-	-	Surface alkylation	Silane coupling agent	[127]
PBAT	Kenaf	PBAT/KF	Asia	D	-	1–5	-	-	-	[128]
PBAT	Linum	PBAT/F	Europe	D		1	-	-	-	[129]
Mater-Bi^®^	Agave	MB/AF	South America	D	-	4–6	-	-	-	[130]

(*) D: discontinuous; C: continuous.

Most of the natural fillers employed generally come from agricultural residues or from low environmental impact collections. Naturally, although most natural fibers are short, their properties—such as diameter, length, and morphology—significantly influence their effectiveness as reinforcements. Consequently, natural fibers are increasingly subjected to preliminary characterization and, in some cases, to treatments to enhance their adhesion to the polymer matrix before use. For instance, bamboo fibers [98,99] have been chemically treated by immersing them for 4 h in an aqueous solution containing 2% NaOH. Subsequently, they were rinsed with distilled water to remove any remaining impurities. Prior to processing, the fibers were oven-dried to eliminate residual moisture.

Flax [102,103,104,105,129] and kenaf [108,109,110,111,112,128] fibers are widely employed in composite manufacturing, either untreated or after undergoing chemical or physical modifications. Notably, kenaf fibers were oven-dried for 24 h at 80 °C to remove excess moisture and then stored in polyethylene bags to prevent water vapor absorption [110]. Subsequently, 40 g of dried fibers were reacted with acetic anhydride in the presence of pyridine as a catalyst, under reflux at 145 °C for different reaction times. After the reaction, the fibers were washed with distilled water and 80% ethanol to remove residual reagents and then dried in a vacuum oven at 60 °C overnight.

Similarly, sisal [114,115,116,117,123] fibers have been broadly adopted in the production of composite materials due to their favorable mechanical and morphological characteristics. In most cases, the fibers have been employed without any pretreatment or with simple pretreatment involving water to remove dirt and pith from the fiber surface, followed by sunlight drying for 48 h to remove moisture [114]. In this latter case, before processing with the matrix, the fibers were heated to 80 °C in a ventilated environment for 6 h.

Hemp [115,119,122,127] fibers also represent a well-established reinforcement in the field of polymer composites. In this case as well, some examples report the use of fibers subjected to pretreatment prior to processing. In a recent study [127], hemp fibers have been subjected to a two-step treatment. In the first step, an alkali treatment was performed by immersing the fibers in a 10% NaOH solution at 10 °C for 1 hour, followed by washing and oven-drying at 80 °C in preparation for the subsequent step. In the second step, the fibers were treated with a coupling agent to enhance the interfacial properties.

Beyond widely used fibers such as jute [103], numerous studies have explored less conventional, locally available alternatives aimed at valorizing regional agricultural residues. Examples include agave [97,130], date palm [118], cannabis sativa [126], pineapple leaf [81], and other location-specific sources [96,100,113,120,121,125]. These fibers can be employed in their untreated form. However, several works also report the adoption of pretreatment or surface-modification steps to enhance the compatibility and performance of alternative fibers within composite systems. For instance, elephant grass fibers were subjected to a mercerization treatment [101]. The treatment involved immersing 200 g of elephant grass fibers in a 10% NaOH solution for 6 h at 70 °C under continuous shaking and stirring, followed by neutralization with 50% acetic acid to remove any absorbed alkali. Finally, the fibers were thoroughly washed with deionized water. Moreover, a bleaching treatment was performed using hydrogen peroxide at room temperature, followed by thorough washing with distilled water and drying in an oven at 50 °C. Moreover, *Himalaya calamus* falconeri fibers were isolated from the culms using a water-retting process followed by mechanical extraction [107]. In this study, a surface modification using a NaOH solution for 5 h at 30 °C was performed to investigate the effect of the alkali treatment on the properties of the biocomposites. In addition, alpha fibers were treated using an in-house optimized method [125]. The fibers were treated with 2% NaOH in water for one hour and then washed several times with distilled water containing 1% acetic acid to neutralize the sodium hydroxide. Finally, they were washed with distilled water until a neutral pH was achieved and dried in an oven at 80 °C for 6 h.

#### 3.1.3. Cellulose and Derivatives

Compared to agricultural and agrifood wastes and natural fibers, only a few studies have focused on cellulose and its derivatives. However, cellulose and lignin are attractive renewable resources that could be employed for the production of biocomposites since they are the main by-products of the pulp and paper industry [46,47,131,132]. Table 3 summarizes the studies available in the literature regarding the use of cellulose and its derivatives as fillers in biocomposites.

**Figure 6:**
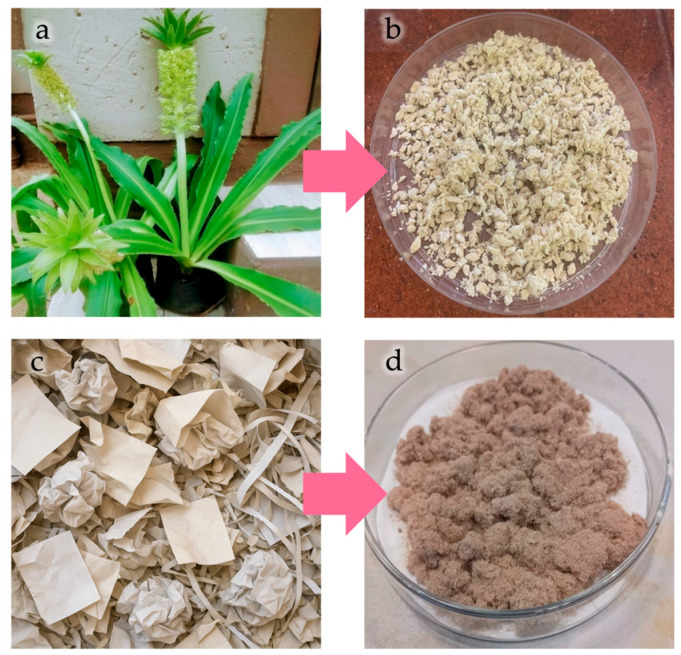
Cellulose extraction from *Eucomis autumnalis* leaves (**a**,**b**) and from wastepaper residues (**c**,**d**). Reprinted (adapted) with permission from [134,137].

Nevertheless, these limited studies have explored cellulose extracted from a diverse range of sources, including the following: *Eucalyptus grandis* [133], *E. autumnalis* (see Figure 6a,b) [134], corn cob [135], residues from the paper and paperboard industry (see Figure 6a,b) [136,137], durian husk [138], agricultural waste [139], *Luffa cylindrica* [140], black liquor [141], softwood almond shells [142], potato peel [92], and coconut fiber [143]. In other cases, commercial cellulose has been used [144].

**Table 3:** Matrices, fillers, and treatments of biocomposites based on cellulose and derivative waste.

Matrix	Cellulose Type	Sample Code	Area	Mechanical Treatment	Drying Temp. [°C]	Drying Time [h]	Other Treatments	Additive	Ref.
PLA	Nanocrystal (eucalyptus)	PLA/CNC	Africa	Freeze-dried	-	-	-	-	[133]
PLA	Eucomis autumnalis	PLA/EA	Africa	Ground and sieved	-	-	Sodium chloride	-	[134]
PLA	Lignin (corn cob)	PLA/Lignin	Asia	-	80	12	-	Plasticizer	[135]
PLA	Micro-fibrillated	PLA/MFC	Europe	-	-	-	-	-	[144]
PLA	Nanocellulose (agro-industrial)	PLA/NCs	South America	-	-	-	Bleaching	-	[136]
PLA	Waste paper	PLA/WP	Europe	UC mill and sieved	60	24	Fatty acid ester	-	[137]
PCL	Microcrystalline (durian rind)	PCL/MCC	Asia	Ground and sieved	100	24	Alkaline and bleaching	-	[138]
PCL	Cellulose (agricultural)	PCL/CNC	Asia	Ground and sieved	-	-	-	-	[139]
PCL	Nanocrystal (luffa cylindrica)	PCL/MLW	South America	-	-	-	Acid and bleaching	Compatibilizer	[140]
PBAT	Lignin (black liquor)	PBAT/lignin	Europe	-	80	12	-	-	[141]
PBAT	Microcrystalline (almond shells)	PBAT/as-MCC	Europe	Ground and sieved	80	4	-	-	[142]
PVA	Cellulose acetate (potato peel)	PVA-CA/Starch	Asia	Ground and sieved	-	-	-	-	[145]
PVA	Nanofibrils (coconut)	CCNF/PVA	Asia	-	-	-	Alkaline and bleaching	-	[143]

UC: ultra-centrifugal.

Mechanical treatments typically involved grinding and sieving [134,138,139,142,145] and in most cases, oven-dried at temperatures between 60 and 100 °C for 12–24 h, prior to processing. Moreover, in many cases, it was subjected to treatments that included sodium chloride [134], bleaching [136], a combination of alkaline and bleaching [138,143], and fatty acid ester [137] to improve its compatibility with polymer matrices. Plasticizers [135] or compatibilizers [140] were also used.

#### 3.1.4. Alternative Plant-Derived Waste

Recently, although still relatively limited, filler research has increasingly focused on exploring alternative plant-derived wastes as sustainable reinforcements in biocomposites (see Table 4).

**Figure 7:**
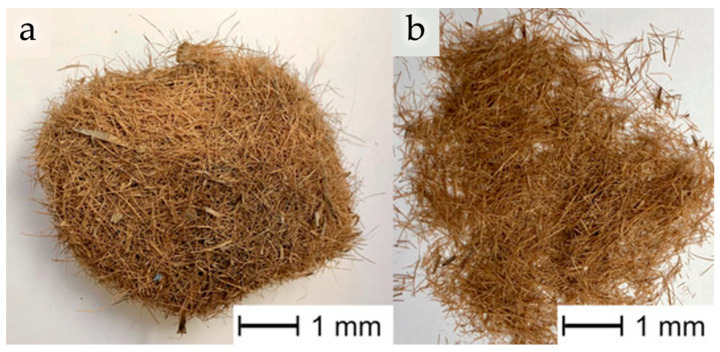
Images of as-received fibrous ball of *Posidonia oceanica* (*Egagropili*) (**a**) and *Posidonia oceanica* fibers after the cleaning process (**b**). Reprinted (adapted) with permission from [147].

*Posidonia oceanica* (see Figure 7) has been incorporated as a filler with the dual purpose of mitigating disposal problems and enhancing valorization opportunities [146,147,148,150].

Moreover, some fillers, such as aloe [149], algae [151], and others [152,153], were employed as fillers taking advantage of the presence of intrinsic compounds that can exert a plasticizing effect. As reported in Table 4, in many cases, mechanical treatments were applied, such as grinding and sieving, followed by drying at temperatures between 60 and 90 °C for 12–24 h.

### 3.2. Animal-Derived Waste

Although most studies have focused on plant-derived fillers, animal-derived organic waste has recently gained growing attention as a valuable resource for developing biodegradable and functional biocomposites. Large quantities of residues are generated annually from the fish, shellfish, poultry, and livestock sectors, including bones, shells, eggshells, feathers, and wool fibers. These by-products contain minerals and proteins, such as calcium carbonate, collagen, chitin, and keratin, which exhibit distinctive chemical compositions and morphologies.

**Figure 8:**
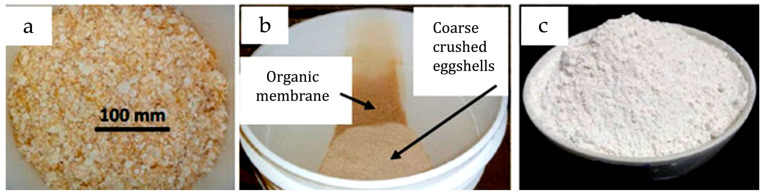
Semi-crushed eggshells (**a**), washing/rinsing step (**b**), and finely ground eggshell powder (**c**). Reprinted (adapted) with permission from [154].

Relevant examples of biocomposites based on animal derived filler are listed in Table 5. Among them, numerous formulations based on PLA and eggshell powders are reported (see Figure 8) [154,155,156]. Eggshells are mainly composed of calcium carbonate and minor amounts of organic proteins. Up to 10% waste eggshell was successfully incorporated into a PLA matrix [154]. Moreover, marine residues such as oyster [81], crab [157], or mollusk [158,159] shells—rich in chitin, chitosan, and calcium salts—have been successfully added to PLA or PHA matrices. In the case of shell powders, additional treatments such as calcination, acid–base purification, or deproteinization are often applied to remove residual impurities and to improve interfacial adhesion with the polymer matrix [157,158].

Fish-processing wastes, such as bones [161], scales [159], or gelatin [160], containing hydroxyapatite or collagen, and other nitrogen-based molecules, have been used as fillers in a PLA- and Mater-Bi^®^-based composite. In addition, wool waste powder, obtained from sheep fur not suitable for textile production, was added to the PLA solution as a functional filler [162]. Wool is rich in keratin proteins and can be processed in fibrous or powdered form.

Chicken feathers and silk waste represent two additional protein-based animal-derived fillers that have recently attracted increasing interest for biodegradable biocomposites. Chicken feathers, mainly composed of keratin, are generated in large quantities by the poultry industry and have been processed in milled or chopped form prior to incorporation into PLA matrices [159,163]. Silk-derived waste, consisting of fibers, powders, nanoparticles, or nanofibers, represents a particularly versatile class of protein-based fillers. Waste silk has been successfully incorporated into PLA [164,165,166], PBS [169,170], PBAT/PLA [167], and PLA/PCL [168] matrices. In some cases [166,167], compatibilizers were added aiming to further enhance interfacial adhesion and performance of the composite.

Similar to what has been observed with plant-based waste, animal wastes generally undergo drying, grinding, and sieving before being used as fillers. The resulting powders or fibers exhibit a wide range of densities, particle sizes, and surface chemistries, which influence their processability and dispersion within polymeric matrices.

## 4. Processing, Properties Application, and End of Life of the Natural-Waste-Based Biocomposites

The performance of natural-waste-based biocomposites strongly depends on the correlation between filler characteristics, processing parameters, and the adopted manufacturing technique. In this section, the main processing routes, the resulting mechanical and functional properties, and the application fields of these materials are critically reviewed. Particular attention is given to how the intrinsic nature of each waste-derived filler influences composite performance and end-of-life behavior, including biodegradation and disintegration.

### 4.1. Plant-Derived Organic Waste

#### 4.1.1. Agricultural and Agrifood Waste

The use of agricultural and agro-industrial residues as fillers in biocomposites represents a promising strategy to enhance material performance while providing environmental and economic benefits. 

As summarized in Table 6, several studies have investigated the incorporation of these wastes into different polymer matrices. Undoubtedly, PLA is the most commonly used matrix, together with commercial blend Mater-Bi. It is followed by PCL, PBS, PHA, PHB, and, finally, PBAT. In most cases, melt compounding (MC) was used in combination with various molding processes—compression [58,62,66,75,85,86,90], injection [52,56,57,59,67,70,76,78,79,80,84,87,91,95], and hot process [51,74,81,82,83]—while some studies used 3D printing to manufacture filaments or complex structures [53,54,58,60,61,64,65,68,77,92,93,94]. In very few cases, films were obtained using MC combined with film blowing [55] or solvent casting [63,69].

In PLA-based composites, the incorporation of fillers, such as artichoke plant [51], banana [52], hazelnut shells [56,57], *Hedysarum coronarium* [58], mango seed [59], opuntia ficus indica [62], tangerines [70], and rice straws [66,67], using conventional molding techniques has generally led to significant mechanical improvements, including increases in tensile [52] and flexural strength [52,56], and modulus [51,56,58,62]. In some cases, some fillers have also contributed to barrier performance [59] and sustainability [56,70], while offering the advantage of easy scalability [57]. On the other hand, PLA-based composites processed by using 3D printing and incorporation the following filler: cocoa husk and bean [53,54], rice husk and straw [64,65,68], olive wood [60], wheat middling and wastes [72,73], and *Opuntia ficus indica* [60,61] showed an increase in tensile strength [53,64,65,68] stiffness [54], flexural [68] proprieties, a good filler dispersion [72], and good processability [61]. In the case of PLA-based films, through melt compounding combined with film blowing or solvent casting, the fillers involved, such as grape pomace [55], orange peel [63], sesame husk [69], and tomato peel [71], mainly improved the functional and environmental properties, including antioxidant and antimicrobial activity [55], as well as biodegradability [63] and disintegration rate [69].

In PCL-based composites, conventional molding techniques with fillers such as date seed [74], olive stones [74], waste bean [75], and wheat bran [74] mainly improved the modulus and especially the thermal properties, while wheat bran also showed a plasticizing effect. Similarly, PBS-based composites, processed by injection molding or 3D printing with fillers such as apple pomace [76], cocoa bean shells [77], almond shell [78,80], and onion peel [79] have shown improvements in impact and tensile strength, elastic modulus, ductility, and disintegration rate, with some composites exhibiting accelerated biodegradation under soil conditions. Furthermore, the use of compatibilizers has improved ductility and mechanical performance [79,80].

**Figure 9:**
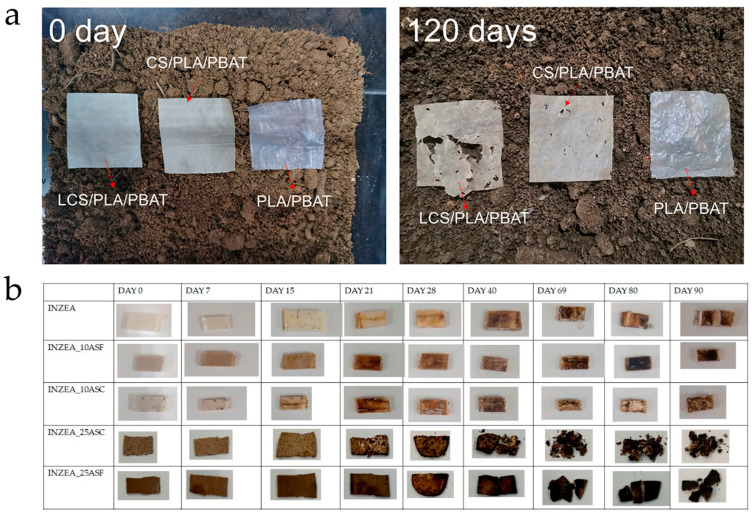
Biodegradability tests of biocomposite film in soil (**a**) and visual appearance of INZEA-based biocomposites with 10 and 25 wt % of ASP at two grinding levels at different testing days at 58 °C. injected ones in composting conditions (**b**). Reprinted (adapted) with permission from [87,95].

Composites based on PHA [81,82], PHB [83], PHBV [84], and PBAT [85,86,87] were mostly processed by hot pressing, compression, or injection molding. These systems showed improvements in terms of mechanical properties, stiffness, thermal stability, permeability, and biodegradability, with several studies confirming the effectiveness of degradation in soil or compost, as shown in Figure 9.

**Figure 10:**
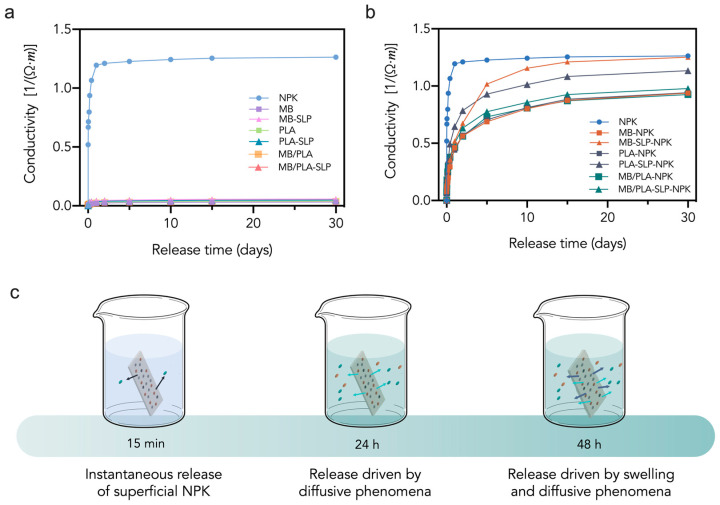
Fertilizer release as function of time for free NPK and 3D-printed devices (**a**,**b**); pictorial description of NPK release mechanism: dots represent NPK powder released in water (**c**). Reprinted (adapted) with permission from [94].

Finally, composites based on commercial blend Mater-Bi^®^ (MB), processed via injection molding or 3D printing with fillers including almond shell [88,89], grape pomace [90], hazelnuts shells [91], *Hedysarum coronarium* [92], *Opuntia ficus indica* [93], and tomato plant [94], exhibited improvements in mechanical properties, ductility, rigidity, and fertilizer release [43], depending on the filler and selected matrix (see Figure 10).

**Figure 11:**
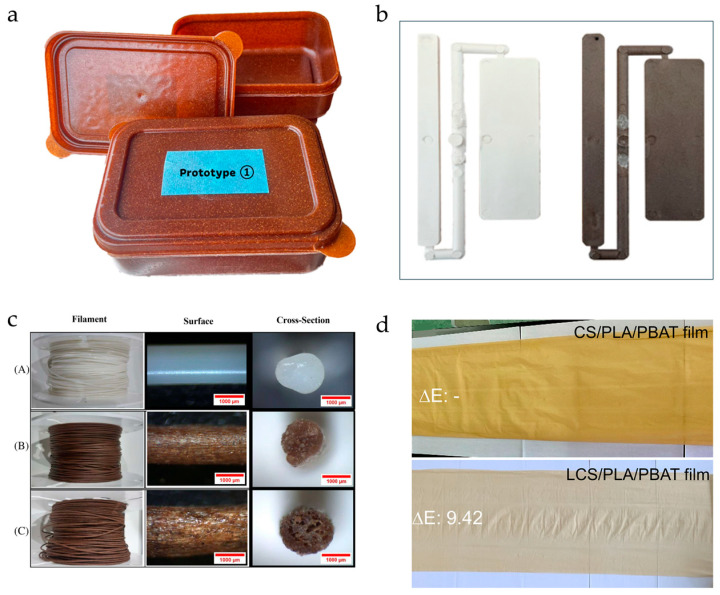
Prototype of biodegradable food containers (**a**) and biocomposites samples (**b**) obtained for injection molding; biocomposite filament for 3D printing applications (A) pure PBS, (B) PBS/10%CBS, (C) PBS/20%CBS (**c**); biocomposite film for food packaging applications (**d**). Reprinted (adapted) with permission from [67,77,87,91].

Mainly, applications were focused on packaging [55,59,62,69,71,74,77,80,81,83,84,85,86,87,88,89] and 3D printing filaments [53,54,61,64,68,72,73,78,90,94], with some studies targeting the automotive [58,65] and industrial [51,56,60,74,75] sectors (see Figure 11).

Specifically, some studies have highlighted additional functionalities, such as fertilizer (NPK) release in MB, PLA and the blend MB/PLA composites based on tomato plant (SLP) filler [93,94], as seen in Figure 9, or increased sustainability, with tests conducted in various environments, including soil burial [63,68,84,88], composting [70,95], and disintegration [69,78,83] studies.

#### 4.1.2. Natural Fibers

The ongoing search for eco-sustainable materials is increasingly leading to the development of biocomposites based on biodegradable polymers and natural fillers. Indeed, the intrinsic reinforcement capacity of the used fillers (see Table 2), maintaining their biodegradability, helps reduce environmental impact and promote the sustainable use of renewable resources. Table 7 summarizes recent studies on these systems, highlighting the relationship between processing, properties, and end-of-life scenarios.

As already seen above, among the various matrices used, PLA remains undoubtedly the most studied, mainly thanks to its biobased origin, good processability, and, above all, its wide availability on the market compared to other new biodegradable polymers.

PLA-based composites obtained by melt compounding followed by compression or injection molding exhibited generally significant improvements in mechanical performance [110,111,112,114,115,116,117]. For example, PLA reinforced with *Abutilon indicum* [96], or bamboo fiber [98,99], showed an increase in tensile and thermal resistance, especially when the filler content and fiber dispersion were optimized. Moreover, PLA reinforced with elephant grass [101], flax [103,104], and Falconeri [107] fibers showed an enhanced tensile strength and stiffness, suggesting that high fiber tensile strength and chemical surface treatments are key factors governing stress transfer and interfacial bonding. Indeed, when untreated natural fibers are used, low interfacial adhesion can still limit the efficiency of reinforcement [100]. On the other hand, several studies have explored the use of these biocomposites in additive manufacturing. In particular, PLA reinforced with agave [97] and kenaf [108] fibers processed by 3D printing showed improved impact strength and stiffness, making these systems suitable candidates for functional components and filaments for fused deposition modeling (FDM). Similarly, PLA coated with continuous flax fibers (CFY) resulted in an increase in tensile modulus and, especially, structural integrity of printed parts [102]. Lower rotation speed during extrusion could improve the fiber aspect ratio, leading to an improvement in the mechanical properties of the 3D-printed filament [105]. Consequently, parameter control can be as crucial as the filler choice in optimizing the performance of printed biocomposites.

In addition to mechanical performance, some works have investigated the environmental impact of the end-of-life cycle of these systems [109]. For example, PLA reinforced with elephant grass [101] and hemp shives [106] showed an increase in biodegradation rate under soil conditions, while the incorporation of *Pennisetum setaceum* [113] led to accelerated composting behavior. This indicates that some natural fillers can act as nucleating sites for microbial attack or moisture diffusion, thus promoting biodegradation. Nonetheless, despite these promising results, the end-of-life aspect remains unexplored for most PLA biocomposites.

Besides PLA, other biodegradable polymers such as PCL, PBS, PHA, PHB, PHBV, and PBAT have also been reinforced with natural fibers. For example, PCL-based composites with date palm [118,120] and hemp fiber [119] showed significant improvement in tensile and flexural strength, suggesting that the flexibility of PCL can be counterbalanced through the addition of stiff fibrous reinforcements. Similarly, PBS composites with *Curaua* [121] and hemp fibers [122] showed improved mechanical performance and, especially for hemp fiber, good sustainability due to their biodegradation under enzymatic hydrolysis and soil-burial conditions.

Particularly interesting are the results obtained on PHB and PHVB composites using *pineapple leaf* [81], sisal [123], and alpha fibers [124], where not only were tensile strength properties improved, but also good recyclability and degradability were observed when subjected to water absorption in distilled and seawater. On the contrary, PBAT-based composites have been mainly developed for packaging applications. For example, PBAT combined with hemp through solvent casting showed antioxidant and antimicrobial activity, confirming its potential use in active packaging [127]. On the other hand, PBAT reinforced with kenaf [128] or alternative [125,126,129] fibers showed improved mechanical performance and biodegradability, especially when compatibilizers were added. 

**Figure 12:**
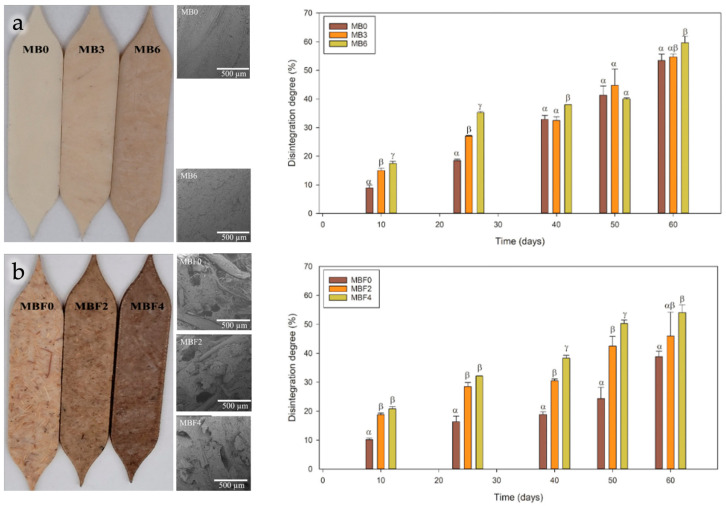
Pure Mater-Bi^®^ (MB, **a**) and agave based biocomposites samples (MBF, **b**) together with their respective disintegration rate at different reprocessing cycles. Letters (α, β and γ) indicate significant differences. Reprinted (adapted) with permission from [130].

The incorporation of agave fibers into Mater-Bi^®^ matrix, processed by melt compounding and compression molding, resulted in composites with enhanced recyclability and compostability (see Figure 12) [130].

#### 4.1.3. Cellulose and Derivatives

Following the analysis of agricultural and natural-fiber wastes, a more limited number of studies have investigated the use of cellulose and its derivatives as fillers in biodegradable matrices. Despite their smaller presence in the literature, these works demonstrate the strong potential of cellulose structures to produce mechanically reinforced and sustainable composites. The main formulations, processing routes, and applications are summarized in Table 8.

**Figure 13:**
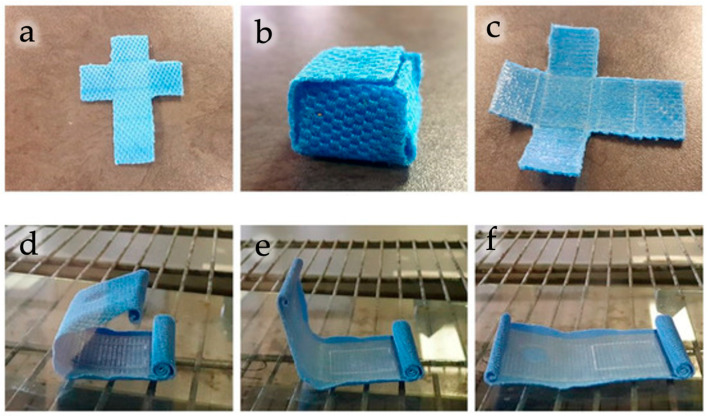
PLA/CNC bionanocomposite prototypes showing the material behavior before, during, and after heat-activated shape memory response: (**a**–**c**) cross-patterned prototype; (**d**–**f**) grid-patterned prototype. Reprinted (adapted) with permission from [133].

PLA-based composites containing cellulose nanocrystals (CNCs) or micro-fibrillated cellulose (MFC) exhibited significant improvements in mechanical strength, elasticity, and shape-memory behavior. More in detail, PLA/CNC composites fabricated by 3D printing exhibited marked improvements in mechanical strength and shape-memory behavior (see Figure 13), confirming the effectiveness of cellulose nanocrystals as reinforcing and functional agents [133]. PLA/MFC films have been processed via film blowing, for flexible packaging applications, and enhanced mechanical properties compared with neat PLA were observed [144]. In addition, composites filled with nanocellulose from agricultural sources [136] displayed stable morphological structures and increased tensile strength and stiffness, confirming the efficiency of cellulose as a reinforcing agent. Similarly, *Eucomis autumnalis* cellulose has been incorporated into PLA by compression molding, obtaining composites with homogeneous filler dispersion and stable tensile performance that allow the production of good-quality filament for 3D printing applications [134].

The introduction of lignin in PLA [135] produced composites with higher toughness and elongation at break, ideal for biomedical applications. Additionally, waste-paper cellulose [137] was successfully used to obtain biocomposites with improved mechanical integrity and sustainability, demonstrating the potential of secondary cellulose streams.

The addition of agriculture waste-derived cellulose to PCL led to the enhancement of the mechanical properties of the materials [138,139,140]. When lignin is added to PBAT matrices, it enhances the modulus while also promoting photo-degradability, a desirable property for packaging and outdoor exposure [141,142]. Coconut nanofibrils and potato-peel cellulose acetate incorporated into PVA-based systems enhanced mechanical properties and biodegradability, confirming the suitability of cellulose derivatives for single-use packaging [143,145].

Regarding the end of life, cellulose-reinforced systems generally exhibited accelerated disintegration under composting and soil-burial conditions [138,145]. The hydrophilic nature and high surface reactivity of cellulose promote hydrolytic and enzymatic degradation, enabling environmentally compatible disposal.

#### 4.1.4. Alternative Organic Waste

Research has recently expanded toward the use of less conventional plant-derived wastes. These materials, often coming from marine or desert ecosystems, further expand the range of renewable resources that can be valorized in biodegradable polymer matrices. The main examples, together with processing methods, properties, and related applications, are summarized in Table 9.

**Figure 14:**
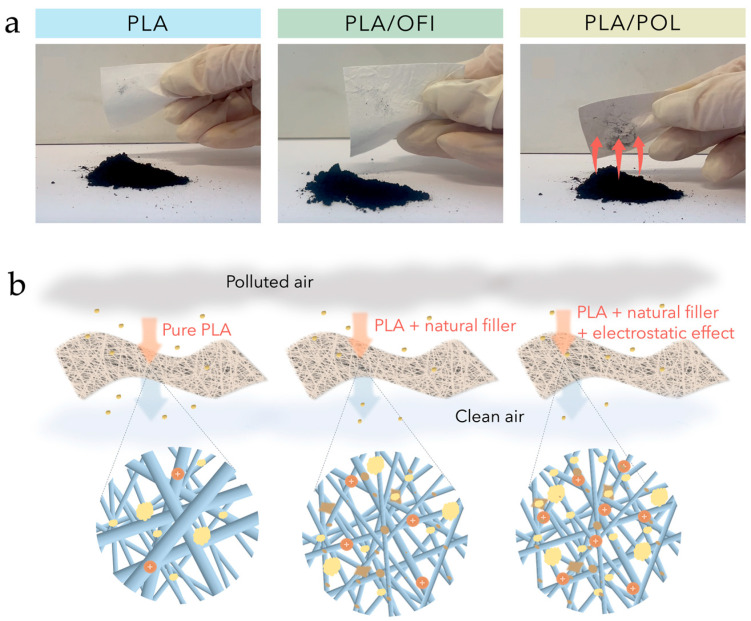
Carbon powder adsorbed on PLA, PLA/OFI, PLA/POL by electrostatic interactions, red arrows represent improved electrostatic attraction conferred by lignin powder (**a**); air filtration mechanism of PLA/POL membranes (**b**). Reprinted (adapted) with permission from [148].

*Posidonia oceanica* residues, available as leaves or fibrous balls, were typically incorporated into PLA or Mater-Bi^®^ matrices by compression or injection molding, producing composites with good processability, improved ductility, and enhanced sustainability [146,147,150]. When used as a powder dispersed in PLA solution and processed via electrospinning [148], this filler allowed the generation of highly porous structures with excellent mechanical strength, improved electrostatic attraction, and filtration efficiency (see Figure 14a,b), producing a device suitable for air-purification applications.

Other natural sources, such as aloe vera [149] and microalgal biomass [151], were processed via injection molding or extrusion, exploiting the presence of bioactive compounds that act as natural plasticizers and photo-protective agents. Aloe vera-based composites exhibited greater UV resistance and accelerated biodegradation under compost or soil conditions. From an end-of-life perspective, microalgal-based composites demonstrated accelerated disintegration and biodegradation under composting or soil-burial conditions.

Similarly, *Moringa oleifera* [152] and babassu [153] residues were combined with PBAT and PBAT/PHB matrices through wire-extension techniques, producing flexible and transparent films with improved mechanical and barrier properties while maintaining full biodegradability. These characteristics represent an appealing feature for food packaging and mulch films.

This confirms that even unconventional plant residues can be effectively used as active fillers in biodegradable polymers, increasing their functional properties, without compromising their environmental performance.

### 4.2. Animal-Derived Organic Waste

In line with what has been observed for plant-derived fillers, animal-derived residues have also been successfully integrated into biodegradable polymer matrices to obtain sustainable and functional composites. The use of such fillers not only promotes the valorization of organic by-products from the fishery sectors but also enables the development of materials with unique combinations of mechanical and functional properties. The main examples, together with processing routes and applications, are reported in Table 10.

After the selection and processing of animal-derived fillers, biodegradable composites have been developed to evaluate their performance and application. In these systems, residues such as eggshell, marine shells, fish bones, gelatin, and wool waste were incorporated into different polymeric matrices using techniques including melt compounding, injection molding, compression molding, film blowing, and 3D printing.

**Figure 15:**
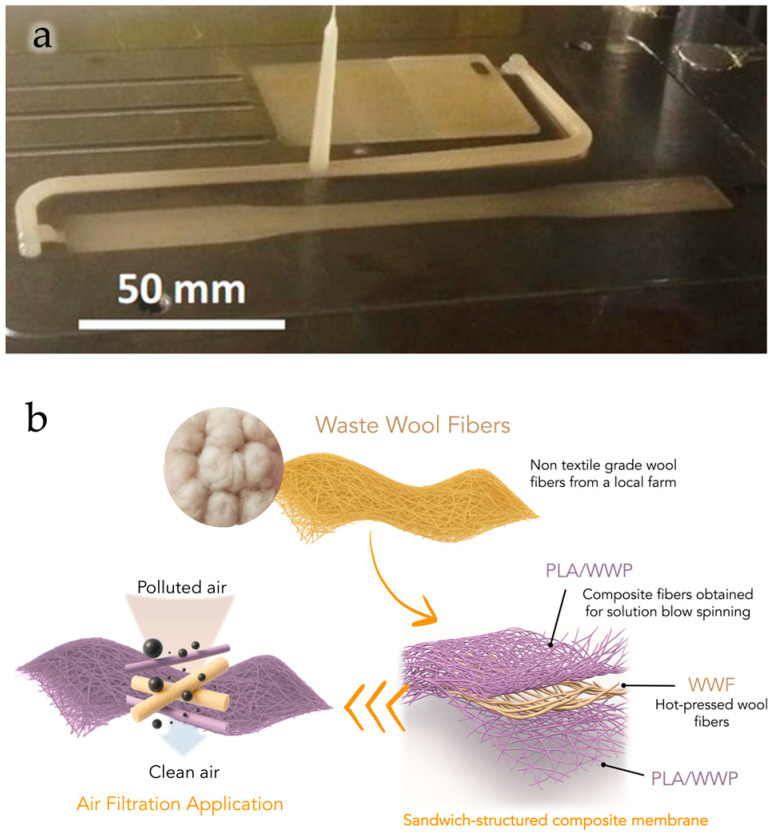
Injection-molded specimens of PLA and eggshells biocomposites (**a**); sandwich-structured membranes based on waste wool fibers (WWFs) produced by combining a central layer of hot-pressed wool fibers with two layers of fibrous membrane based on PLA and waste wool powder (WWP) obtained by the solution blow spinning technique, and tested for air filtration application (**b**). Reprinted (adapted) with permission from [154,162].

More in detail, eggshell-based composites, prepared mainly through melt compounding and injection molding (see Figure 15a), generally show an increase in stiffness due to the high calcium carbonate content [154,155,156]. Shellfish-derived fillers, such as oyster or crab shells, also act as reinforcing agents, improving the mechanical properties and enhancing biodegradability of the resulting materials [81,157,158,159]. Fish-derived fillers, including bones, scales, and gelatin residues, provide protein- and mineral-rich phases that contribute to improved biodegradability [159], processability and mechanical properties [161], or barrier and antioxidant properties [160], which are particularly beneficial for packaging and food-contact applications. More in detail, when compared to the pure PLA, fish scale-based composites showed superior flexural properties (+10% strength, +32% modulus) and moderate biodegradation. On the other hand, seashell-based composites excelled in thermal stability and impact resistance (+25%), confirming their suitability for durable applications [159].

Chicken feather reinforced PLA composites, processed by injection or compression molding, showed improved sustainability and enhanced biodegradation under soil conditions, while maintaining adequate mechanical properties for agricultural applications [159,163]. The keratin-rich nature of feathers promotes soil degradation, making these systems particularly attractive for short-life and environmentally exposed products [159]. If compared with the neat PLA matrix, chicken feather-based composites achieved 4.12% higher tensile strength and rapid biodegradation (8.85% weight loss in 30 days), ideal for short-life products [159].

Silk-based composites exhibit an even broader technological versatility. Depending on their form (fibers, powders, nanoparticles, or nanofibers) and on the selected polymer matrix (PLA, PBS, PBAT/PLA, PLA/PCL), silk-reinforced composites have been processed via injection molding, compression molding, and hand layup. These systems generally show significant improvements in mechanical properties [164,165,166,167,168,169,170], wear resistance [164] (fundamental for textile applications), and thermal stability [168,169,170], with several studies also reporting enhanced physiological and hydrolytic degradation [166,168]. More in detail, the presence of both silk nanoparticles (NP) and nanofibers (SF) reduced the thermo-mechanical degradation of PLA/PCL matrix during the mechanical recycling. The composites showed higher values of thermal stability, barrier properties, microhardness, and impact strength than the neat PLA/PCL blend. The use of NP (compared to SF) led to a significant improvement of the mentioned properties, which was attributed to the better intermolecular interaction of NP with the matrix.

Furthermore, wool waste powders, obtained from non-textile sheep breeds and incorporated in PLA solution, enabled the fabrication of fibrous biocomposite membranes via solution blow spinning with excellent mechanical performance and air filtration efficiency (see Figure 15b), demonstrating their suitability for environmental remediation and air purification systems [162]. In particular, the wool-based membranes exhibited excellent fine particulate removal efficiencies of 99.5% and pressure drops of 70 Pa at a flow rate of 32 L/min. Moreover, the membrane maintained filtration stability up to five reuse cycles and durability under high humidity conditions.

From an end-of-life perspective, most composites containing animal-derived fillers demonstrated enhanced biodegradation and disintegration rates, mainly due to the presence of mineral- or protein-based components that facilitate hydrolytic and enzymatic degradation. Systems incorporating shell powders showed improved compostability and soil disintegration, as the calcium carbonate and chitin phases act as nucleating and hydrolysis-promoting agents [81,158]. Composites based on fish-derived fillers, such as gelatin, underwent accelerated degradation under composting conditions, confirming their potential for eco-friendly disposal [160].

Overall, composites reinforced with animal-derived organic waste exhibit enhanced mechanical and functional properties, often accompanied by increased biodegradability and sustainability. These materials can be valorized for the production of advanced applications composites ranging from biomedical and packaging uses to air filtration and environmental protection, offering an effective strategy to valorize residues from the fishery and livestock sectors within a circular and low-impact materials framework.

### 4.3. Compatibilization Strategies in Plant- and Animal-Derived Biocomposites

Interfacial engineering plays a critical role in determining the final performance of biocomposites, especially when natural waste-derived fillers are incorporated. In most cases, plant- and animal-based fillers exhibit hydrophilic surfaces rich in hydroxyl, carboxyl, or protein-derived functional groups, whereas the most common biodegradable polymer matrices (e.g., PLA, PBAT, PCL, PHAs) are hydrophobic. This intrinsic chemical mismatch often results in weak interfacial adhesion, poor stress transfer, suboptimal mechanical reinforcement, and potential moisture-induced degradation at the interface [171,172]. 

For these reasons, compatibilization strategies are essential to enhance matrix–filler interactions, improve dispersion, reduce interfacial defects, and ultimately enable the development of high-performance biodegradable composites. Various approaches have been proposed in the literature, including surface treatments applied directly to the filler as well as the incorporation of coupling agents or compatibilizers within the polymer matrix. A summary of the most relevant and widely used strategies is reported below.

In biocomposites that use plant- and animal-derived waste, compatibilization is often necessary to improve interfacial adhesion and stress transfer efficiency between the organic filler and the polymer matrix. In this regard, some studies [64,65,66,67,70,71,76,78,79,80,81,98,99,101,110,114,123,124,127,128,137,139,141,147,157] have adopted strategies that can help improve adhesion, dispersion, and, especially, the performance of these systems. Usually, these include the use of coupling agents [64,66,67,70,76,78,80,127,147], compatibilizers [79,80,128,141], and surface treatments applied directly to the natural filler [65,71,81,98,99,101,110,114,124,127,137,139,141,157]. Generally, surface treatments are used to remove impurities, clean the fiber surface, reduce hydrophilicity, or increase surface roughness. In fact, even simple or mild treatments can improve processing and thus lead to more uniform fiber dispersion within composites. For this reason, some work indicates that such interfacial improvements can influence the mechanical performance and processing behavior of these systems. On the other hand, the use of coupling or compatibilizer agents can help to reduce chemical and physical differences between the organic filler and polymer matrix, supporting stronger interfacial adhesion and helping to minimize issues related to moisture or limited stress transfer. However, these strategies are often combined or adopted depending on the type of matrix, the filler used, and the desired properties, offering different ways to improve the compatibility of biocomposites containing organic waste of plant- or animal-derived.

## 5. Conclusions

The valorization of plant- and animal-derived organic waste as functional fillers in biodegradable polymeric matrices represents a consolidated and rapidly expanding research direction. The studies analyzed that waste materials (lignocellulosic residues, natural fibers, cellulose derivatives, or animal by-products) can significantly enhance the mechanical, thermal, functional, and biodegradation behavior of biocomposites, often enabling additional benefits such as antioxidant activity, barrier improvements, fertilizer release, or air-filtration performance. Across all polymer matrices, the effectiveness of these fillers is strongly governed by their intrinsic composition, morphology, and surface chemistry. Grinding, drying, sieving, chemical modification, and compatibilization play a central role in optimizing dispersion and interfacial adhesion. Moreover, the increasing integration of waste fillers into 3D printing and advanced processing technologies confirms their suitability for high-value and technologically relevant applications.

The analysis of recent studies reveals several converging trends in the development of biodegradable biocomposites reinforced with plant- and animal-derived organic waste. First, across all polymer systems, biocomposites generally exhibit improved stiffness, barrier properties, and, in many cases, accelerated biodegradation, confirming the potential of organic waste as functional additives rather than simple fillers. At the same time, the integration of waste valorization within circular-economy models and the expansion toward advanced applications (packaging, additive manufacturing, agriculture, biomedical tools, and environmental remediation) indicate a clear shift from traditional waste management approaches to a materials-engineering perspective. Moreover, the literature consistently shows that filler composition, particle morphology, and surface chemistry strongly govern interfacial adhesion and thus the mechanical and functional properties of the final materials. Surface treatments, compatibilization strategies, and optimized dispersion protocols are emerging as essential tools to overcome the intrinsic incompatibility between hydrophilic fillers and hydrophobic biodegradable matrices.

Despite these promising advances, several challenges remain. The intrinsic variability of waste streams in composition and particle size, and the difficulty in achieving consistent large-scale processing, still limit industrial adoption. Economic factors, including the cost of fine milling, compatibilization agents, and reproducibility, can also represent obstacles for translating laboratory-scale successes into commercial products. Furthermore, the long-term durability regulatory requirements for food-contact applications, and the environmental impact of specific treatments (e.g., alkali or solvent-based processes) require further investigation.

Future research should therefore focus on the following:

i. Optimize filler selection, compatibilization routes, and processing conditions;
ii. Developing scalable and low-energy pretreatment technologies;
iii. Conducting systematic life-cycle and techno-economic assessments to evaluate the true sustainability and industrial feasibility of these systems.

Addressing and overcoming these challenges will be crucial to enable the next generation of high-performance biodegradable biocomposites and to fully unlock the potential of organic waste within a circular-economy framework.

Overall, the current evidence highlights a transition toward more advanced and purposeful material design, in which organic waste is no longer a by-product but a strategic resource for developing high-performance biodegradable biocomposites.

## Figures and Tables

**Table 1:** Matrices, fillers, and treatments of biocomposites based on agricultural and agro-industrial waste.

Matrix	Filler	Sample Code	Area	Mechanical Treatment	Drying Temp. [°C]	Drying Time [h]	Other Treatments	Coupling Agent	Ref.
PLA	Artichoke Plants	PLA/AP	Europe	Ground and sieved	60	12	-	-	[51]
PLA	Banana	PLA/BF	Asia	-	90	6	-	-	[52]
PLA	Cocoa Bean Shell	PLA/CBSW	Europe	Milled and sieved	60	24	-	-	[53]
PLA	Cocoa Husk	PLA/CH	South America	Knife mill and sieved	60	12	-	-	[54]
PLA	Grape Pomace	PLA/GP	Europe	Milled and sieved	48	72	-	-	[55]
PLA	Hazelnut Shell	PLA/HSF	-	UC mill and sieved	60	24	-	-	[56]
PLA	Hazelnuts Shell	PLA/HSF	Europe	Milled and sieved	60	16	-	-	[57]
PLA	Hedysarum Coronarium	PLA/HC	Europe	Ground and sieved	90	~16 on	-	-	[58]
PLA	Mango Seed	PLA/MS	South America	Milled and sieved	52	24	-	-	[59]
PLA	Olive Wood	PLA/OW	Europe	UC mill and sieved	60	-	-	-	[60]
PLA	Opuntia Ficus Indica	PLA/OFI	Europe	Ground and sieved	90	~16 on	-	-	[61]
PLA	Opuntia Ficus Indica	PLA/OFI	Europe	HS mill and sieved	70	~16 on	-	-	[62]
PLA	Orange Peel	PLA/OPP	Europe	Ground and sieved	60	18	-	-	[63]
PLA	Rice husk	PLA/RH	Asia	Ground and sieved	90	12	-	yes	[64]
PLA	Rice husk	PLA/MNRH	Asia	-	-	-	Alkaline treatment	-	[65]
PLA	Rice Straw	PLA/RS	Asia	Milled and sieved	105	12	-	yes	[66]
PLA	Rice Straw	PLA/RS	Asia	Knife mill and sieved	80	6	-	yes	[67]
PLA	Rice straw	PLA/RSP	Asia	Ground and sieved	60	12	-	-	[68]
PLA	Sesame Husk	PLA/SSHP	Asia	Milled and sieved	50	36	-	-	[69]
PLA	Tangerines	PLA/TPF	Europe	UC mill and sieved	40	48	-	yes	[70]
PLA	Tomato Peel	PLA/THP	Europe	Milled and sieved	60	8	Alkaline extraction	-	[71]
PLA	Wheat Middling	PLA/WM	Europe	Milled and sieved	75	1	-	-	[72]
PLA	Wheat Wastes	PLA/WW	Europe	-	-	-	-	-	[73]
PCL	Date Seed	PCL/DS	Europe	Ground and sieved	-	-	-	-	[74]
PCL	Olive Stones	PCL/OS	Europe	Ground and sieved	-	-		-	[74]
PCL	Waste Bean	PCL/WaB	Europe	-	50	~16 on	-	-	[75]
PCL	Wheat Bran	PCL/WhB	Europe	Ground and sieved	-	-	-	-	[74]
PBS	Apple Pomace	PBS/AP	Asia	Milled and sieved	80	12	-	yes	[76]
PBS	Cocoa Bean Shells	PBS/CBS	South America	Ground and sieved	80	16	-	--	[77]
PBS	Almond Shell Flour	PBS/ESF	Europe	Milled and sieved	50	24	-	yes	[78]
PBS	Onion Peels	PBS/OP	Europe	Knife mill and sieved	60	24	-	-	[79]
PBSA	Almond Shell	PBSA/AS	North America	Ground and sieved	68	48	-	yes	[80]
PHA	Pineapple Leaf	PHA/PL	Asia	Ground and sieved	-	-	Alkaline treatment	-	[81]
PHA	Rice Husk	PHA/RH	Asia	Ground and sieved	105	24	-	-	[82]
PHB	Almond Shell	PHB/AS	Europe	Ground and sieved	60	24	-	-	[83]
PHB	Rice Husk	PHB/RH	Europe	UC mill	60	24	-	-	[83]
PHB	Seagrass	PHB/SG	Europe	UC mill	60	24	-	-	[83]
PHBV	Peach Palm	PHBV/PP	South America	Ground and sieved	60	48	-	-	[84]
PBAT	Corn stover	PBAT/CS	Asia	Ground and sieved	80	12	-	-	[85]
PBAT	Mangosteen	PBAT/M	Asia	Ground and sieved	80	2	-	-	[86]
PLA/PBAT	Corn Stover	PLA/PBAT/CS	Asia	-	50		-	-	[87]
Mater-Bi^®^	Almond Shell	MB/AS	Europe	Milled and sieved	105	24	-	-	[88]
Mater-Bi^®^	Almond Shell	MB/AS	Europe	Milled and sieved	102	24	-	-	[89]
Mater-Bi^®^	Grape Pomace	MB/GP	Europe	Milled and sieved	80	~16 on	-	-	[90]
Mater-Bi^®^	Hazelnut Shells	MB/HS	Europe	-	60	4	-	-	[91]
Mater-Bi^®^	Hedysarum Coronarium	MB/HC	Europe	Ground and sieved	60	~16 on	-	-	[92]
Mater-Bi^®^	Opuntia Ficus Indica	MB/OFI	Europe	Ground and sieved	90	~16 on	-	-	[93]
Mater-Bi^®^	Tomato Plant	MB/TP	Europe	Ground and sieved	40	~16 on	-	-	[94]
Inzea^®^	Almond Shell	Inz/AS	-	HS mill and sieved	-	-	-	-	[95]

UC: ultra-centrifugal; HS: high speed; on: overnight.

**Table 4:** Matrices, fillers, and treatments of biocomposites based on alternative organic waste.

Matrix	Filler	Sample Code	Area	Mechanical Treatment	Drying Temp. [°C]	Drying Time [h]	Additive	Ref.
PLA	Posidonia Oceanica leaves	PLA/PO	Europe	Ground and sieved	80	12	-	[146]
PLA	*Egagropili*	PLA/POS	Europe	Crushed	65	12	DCP, Plasticizer	[147]
PLA	Posidonia Oceanica leaves	PLA/POL	Europe	Ground and sieved	60	12	-	[148]
PLA	Aloe vera	PLA/AV	S. America	-	60	16	-	[149]
PLA	Posidonia Oceanica leaves	PLA10A	Europe	Ground and sieved	90	12	-	[150]
PLA	Duneleila Tertiolecta algae	PLA/AB	Asia	-	-	-	-	[151]
PBAT	Moringa oleifera	PBAT/MO	S. America	Ground and sieved	60	24	-	[152]
PBAT/PHB	Babassu	PBAT/PHB/BS	Europe	Ground and sieved	60	20	-	[153]

DCP: Dicumyl peroxide.

**Table 5:** Matrices, fillers, and treatments of biocomposites based on animal waste.

Matrix	Filler	Sample Code	Area	Mechanical Treatment	Drying Temp. [°C]	Drying Time [h]	Other Treatments or Additive	Ref.
PLA	Eggshell	PLA/WE	N. America	Ground	80	4	-	[154]
PLA	Eggshell	PLA/ESP	Asia	Ground	-	-	-	[155]
PLA	Eggshell	PLA/WES	N. America	Ground	-	-	-	[156]
PLA	Fish gelatin	PLA/FG	Europe	-	80	12		[160]
PLA	*P. undulata* shell	PLA/PUS	Asia	Ground	100/60	24/48	Calcination	[158]
PLA	Crab shells	PLA/CSP	Asia	Ground	60	12	HCl, NaOH	[157]
PLA	Anchovy fish bone	PLA/EE	Europe	Ground	60	12	-	[161]
PLA	Fish scales	PFS:PLA	Asia	Milled	60	24	-	[159]
PLA	Seashells	PSS:PLA	Asia	Milled	60	24	-	[159]
PLA	Wool	PLA/WP	Europe	Ground	60	12	-	[162]
PLA	Chicken feathers	PCF:PLA	Asia	Milled	60	24	-	[159]
PLA	Chicken feather	CCF/PLA	Asia	Cut	60	6	-	[163]
PLA	Silk fibers	PLA/SF	Asia	Chopped	-	-	-	[164]
PLA	Silk fibers	WSF/PLA	Asia	Chopped	-	-	-	[165]
PLA	Silk fibers	PLA/Silk	Europe	-	-	-	maleic anhydride, ethylene glycidyl methacrylate	[166]
PLA/PBAT	Silk powder	PLA/PBAT/S	Asia	-	80	24	epoxy oligomeric acrylic resins	[167]
PLA/PCL	Silk nanoparticles	PLA/PCL/NP	Asia	-	40	2	-	[168]
PLA/PCL	Silk nanofibers	PLA/PCL/SF	Asia	-	85	2	-	[168]
PBS	Silk fibers	silk/PBS	Asia	Chopped	100	2	-	[169]
PBS	Silk fibers	wsilk/PBS	Asia	Chopped	100	2	-	[170]
PHA	Oyster shell	PHA/OSP	Asia	Ground	-	-	-	[81]
Mater-Bi^®^	Anchovy fish bone	MB/EE	Europe	Ground	60	12	-	[161]

HCl: hydrochloric acid; NaOH: sodium hydroxide.

**Table 6:** Processing, properties application, and end of life of biocomposites based on agricultural and agro-industrial waste.

Sample Code	Processing	Key Results	Application	End of Life	Ref.
PLA/AP	MC + Hot process	↑ Elastic modulus	Variety of Fields	-	[51]
PLA/BF	MC + Inject. mold.	↑ Flexure and tensile strength	-	-	[52]
PLA/CBSW	MC + 3D printing	↑ Rigidity and load resistance	3D printing filament	-	[53]
PLA/CH	MC + 3D printing	↑ Tensile strength	3D printing filament	-	[54]
PLA/GP	MC + Film blowing	↑ Antioxidant and antimicrobial activity	Packaging	-	[55]
PLA/HSF	MC + Inject. mold.	↑ Flexural modulus and sustainability	Building industry	-	[56]
PLA/HSF	MC + Inject. mold.	Easily scalable	-	-	[57]
PLA/HC	MC + Compress. mold. MC + 3D printing	↑ Elastic modulus Excellent printability	- Automotive	- -	[58]
PLA/MS	MC + Inject. mold.	↑ Barrier properties	Packaging	-	[59]
PLA/OW	MC + 3D printing	↑ Porosity	Non-structural	-	[60]
PLA/OFI	MC + 3D printing	Good processability	3D printing filament	-	[61]
PLA/OFI	MC + Compress. mold.	↑ Stiffness	Packaging		[62]
PLA/OPP	Solvent casting	↑ Biodegradation	-	Soil burial	[63]
PLA/RH	MC + 3D printing	↑ Tensile strength and improved adhesion	3D printing filament	-	[64]
PLA/MNRH	MC + 3D printing	↑ Mechanical and thermal properties	Automotive	-	[65]
PLA/RS	MC + Compress. mold.	Improve interfacial adhesion	-	-	[66]
PLA/RS	MC + Inject. mold.	-	-		[67]
PLA/RSP	MC + 3D printing	↑ Flexure and tensile strength	3D printing filament	Soil burial	[68]
PLA/SSHP	Solvent casting	↑ Tensile and thermal properties	Packaging	Soil degradation	[69]
PLA/TPF	MC + Inject. mold.	↑ Biodegradation	-	Compost soil	[70]
PLA/THP	Solvent casting + compress. mold.	↑ Tensile strength	Packaging	-	[71]
PLA/WM	MC + 3D printing	Good filler dispersion	3D printing filament	-	[72]
PLA/WW	MC + 3D printing	↑ Sustainability	3D printing filament	-	[73]
PCL/DS	MC + Hot process	↑ Modulus and thermal stability	Packaging	-	[74]
PCL/OS	MC + Hot process	↑ Modulus and thermal stability	-	-	[74]
PCL/WaB	MC + Compress. mold.	↑ Modulus and thermal stability	Industrial	-	[75]
PCL/WhB	MC + Hot process	Plasticization effect	Industrial	-	[74]
PBS/AP	MC + Inject. mold.	↑ Impact and tensile strength	-	-	[76]
PBS/CBS	MC + 3D printing	↑ Modulus	Packaging	-	[77]
PBS/ESF	MC + Inject. mold.	↑ Disintegration rate	3D printing filament	Disintegration in soil	[78]
PBS/OP	MC + Inject. mold.	↑ Ductile properties (with compatibilizers)	Automotive	-	[79]
PBSA/AS	MC + Inject. mold	↑ Mechanical properties (with compatibilizers)	Packaging	-	[80]
PHA/PL	MC + Hot process	↑ Biodegradation	Packaging	-	[81]
PHA/RH	MC + Hot process	↑ Biodegradation	-	-	[82]
PHB/AS	MC + Hot process	↑ Permeability	Packaging	Disintegration	[83]
PHB/RH	MC + Hot process	↑ Mechanical properties	Packaging	Disintegration	[83]
PHB/SG	MC + Hot process	↑ Mechanical properties	Packaging	Disintegration	[83]
PHBV/PP	MC + Inject. mold.	↑ Biodegradation	Packaging	Soil burial	[84]
PBAT/CS	MC + Compress. mold.	↑ Stiffness	Packaging	-	[85]
PBAT/M	MC + Compress. mold.	↑ Thermal stability	Packaging	-	[86]
PLA/PBAT/CS	MC + Inject. mold.	↑ Mechanical properties	Packaging	Soil burial	[87]
MB/AS	MC + Inject. mold.	↑ Biodegradation	Packaging	Soil burial	[88]
MB/AS	MC + Inject. mold.	↑ Mechanical properties	Packaging	-	[89]
MB/GP	MC + Compress. mold.	Good fertilizer release	3D printing filament	-	[90]
MB/HS	MC + Inject. mold.	↑ Ductility	-	-	[91]
MB/HC	MC + 3D printing	↑ Rigidity	-	-	[92]
MB/OFI	MC + 3D printing	↑ Fertilizer release	-	-	[93]
MB/TP	MC + 3D printing	↑ Mechanical properties	3D printing filament	-	[94]
Inz/AS	MC + Inject. mold.	↑ Mechanical properties	-	Compost soil	[95]

↑ Increasing properties; MC: melt compounding.

**Table 7:** Processing, properties application, and end of life of biocomposites based on natural fiber waste.

Sample Code	Processing	Key Results	Application	End of Life	Ref.
PLA/AI	MC + Inject. mold.	↑ Mechanical Properties	Industrial	-	[96]
PLA/AF	MC + 3D printing	↑ Impact Tensile	3D printing filament	Compost in soil	[97]
PLA/BF	MC + Inject. mold.	↑ Mechanical and Thermal Properties	Variety of fields	-	[98]
PLA/BF	MC + Inject. mold.	↑ Recycling	Variety of fields	-	[99]
PLA/CS	MC + Inject. mold.	Low Interfacial Adhesion	-	-	[100]
PLA/EG	MC + Inject. mold.	↑ Mechanical Properties and Bio	-	Soil degradation	[101]
PLA/CFY	Extr. coating + 3D printing	↑ Mechanical Properties	3D printing filament	-	[102]
PLA/Flax	MC + Compress. mold.	↑ Tensile Strength	-	-	[103]
PLA/FS	Direct Extr. process	↑ Impact and Tensile Modulus	Automotive	-	[104]
PLA/FS	Direct Extr. process	↑ l/d Ratio at Low Rotational Speed	3D printing filament	-	[105]
PLA/HS	MC + Compress. mold.	↑ Biodegradation	-	Soil degradation	[106]
PLA/THF	Direct Inject. mold.	↑ Mechanical and Thermal Properties	Non-structural	-	[107]
PLA/Jute	MC + Compress. mold.	↑ Resistence creep	-	-	[103]
PLA/KF	MC + 3D printing	↑ Toughness	3D printing filament	-	[108]
PLA/KF	MC + Compress. mold.	↑ Mechanical Properties	-	Soil burial	[109]
PLA/KFA	MC + Inject. mold.	↑ Mechanical and Water Resistance	-	-	[110]
PLA/LK	MC + Hot process	↑ Mechanical Properties	-	-	[111]
PLA/WK	MC + Hot process	↑ Mechanical Properties	Automotive	-	[112]
PLA/PS	MC + Inject. mold.	↑ Biodegradation	-	Compost in soil	[113]
PLA/SF	MC + Inject. mold.	↑ Mechanical and Thermal Properties	Non-structural	-	[114]
PLA/SF	MC + Inject. mold.	↑ Impact and Tensile Modulus	-	-	[115]
PLA/SF	MC + Inject. mold.	↑ Mechanical Properties	-	-	[116]
PLA/MS	MC + Inject. mold.	↑ Mechanical Properties	-	-	[117]
PCL/DP	MC + Compress. mold.	↑ Mechanical Properties	-	-	[118]
PCL/HF	MC + Compress. mold.	↑ Mechanical Properties	Packaging	-	[119]
PCL/DP	MC + Compress. mold.	↑ Flexure and Tensile Strength	-	-	[120]
PBS/C	MC + Compress. mold.	↑ Mechanical Properties	Packaging	-	[121]
PBS/HF	MC + Compress. mold.	↑ Sustainability	-	Enzymatic hydrolysis and soil burial	[122]
PHA/PLF	MC + Hot pression	↑ Biodegradation	3D printing filament	Soil burial	[81]
PHB/SF	MC + Compress. mold.	↑ Recycling	-	-	[123]
PHBV/AF	MC + Inject. mold.	↑ Mechanical Properties (with alkali treatment)	-	Aqueous environment	[124]
PBAT/CF	MC + Compress. mold.	↑ Mechanical Properties	-	-	[125]
PBAT/MF	MC + Compress. mold.	↑ Mechanical Properties	-	-	[125]
PBAT/TF	MC + Compress. mold.	↑ Mechanical Properties	-	-	[125]
PBAT/CS	Solvent casting	↑ Antioxidant and Antimicrobial Activity	Packaging	-	[126]
PBAT/HF	Open blending + Hot press	↑ Biodegradation	-	Soil burial	[127]
PBAT/KF	MC + Hot process	↑ Mechanical Properties (with compatibilizers)	-	-	[128]
PBAT/F	MC + 3D printing	↑ Stiffness and Strength	3D printing filament	-	[129]
MB/AF	MC + Compress. mold.	↑ Recycling	-	Compost in soil	[130]

↑ Increasing properties; MC: melt compounding.

**Table 8:** Processing, properties application, and end of life of biocomposites based on cellulose and derivative waste.

Sample Code	Processing	Key Results	Application	End of Life	Ref.
PLA/CNC	MC + 3D printing	↑ Mechanical, shape memory	4D applications	-	[133]
PLA/EA	MC + Compress mold	No significant changes	3D printing filament	-	[134]
PLA/Lignin	MC + 3D printing	↑ Elongation and toughness	Biomedical	-	[135]
PLA/MFC	MC + Film Blowing	↑ Mechanical	Packaging	-	[144]
PLA/NCs	MC + Compress mold	↑ Mechanical	Packaging	-	[136]
PLA/WP	Melt compounding	↑ Mechanical, sustainability	Packaging	-	[137]
PCL/MCC	Melt compounding	↑ Mechanical, biodegradability	-	Soil degradation	[138]
PCL/CNC	Solution casting	↑ Tensile Strength	-	-	[139]
PCL/MLW	Solution casting	↑ Modulus	-	-	[140]
PBAT/lignin	MC + Film Blowing	↑ Modulus, photo degradation	Packaging	-	[141]
PBAT/as-MCC	MC + Compress. mold.	↑ Modulus	-	-	[142]
PVA-CA/Starch	Solution casting	↑ Mechanical, biodegradability	-	Enzymatic degradation	[145]
CCNF/PVA	Solution casting	↑ Mechanical	Packaging	-	[143]

↑ Increasing properties; MC: melt compounding.

**Table 9:** Processing, properties application, and end of life of biocomposites based on alternative organic waste.

Sample Code	Processing	Key Results	Application	End of Life	Ref.
PLA/PO	Compression molding	↑ Sustainability	-	-	[146]
PLA/POS	Injection molding	↑ Ductility, Sustainability	-	-	[147]
PLA/POL	Electrospinning	↑ Mechanical, Sustainability	Air filtration	-	[148]
PLA/AV	Injection molding	↑ UV Resistance	-	-	[149]
PLA10A	Compression molding	↑ Sustainability	-	-	[150]
PLA/AB	Extrusion	↑ Degradation, Sustainability	-	Compostability, soil degradation	[151]
PBAT/MO	Wire extension (film)	↑ Fruit Shelf Life	Packaging	-	[152]
PBAT/PHB/BS	Wire extension (film)	↑ Mechanical, Sustainability	Packaging and mulch films	-	[153]

↑ Increasing properties.

**Table 10:** Processing, properties application, and end of life of biocomposites based on animal waste.

Sample Code	Processing	Key Results	Application	End of Life	Ref.
PLA/WE	Injection Molding	↑ Tensile modulus	-	-	[154]
PLA/ESP	Film Casting	↑ Mechanical, sustainability	Packaging	-	[155]
PLA/WES	Injection Molding	No significant changes	-	-	[156]
PLA/FG	Film Blowing	↑ Oxy barrier, antioxidant	Packaging	Compostability	[160]
PLA/PUS	Compression Molding	↑ Mechanical, sustainability	Packaging, utensils	Soil degradation	[158]
PLA/CSP	3D Printing	↑ Mechanical	Biomedical	-	[157]
PLA/EE	3D Printing	↑ Mechanical, ↑ processability, sustainability	Packaging	-	[161]
PFS:PLA	Compression Molding	↑ Biodegradation	Fishing gears	Soil degradation	[159]
PSS:PLA	Compression Molding	↑ Mechanical, thermal	Thermal insulation	Soil degradation	[159]
PLA/WP	Solution Blow Spinning	↑ Mechanical, sustainability	Air filtration	-	[162]
PCF:PLA	Compression Molding	↑ Mechanical, biodegradation	Sustainable Agriculture	Soil degradation	[159]
CCF/PLA	Injection Molding	↑ Sustainability	-	-	[163]
PLA/SF	Hand Layup	↑ Mechanical, wear prop.	Textile	-	[164]
WSF/PLA	Hand Layup	↑ Mechanical	-	-	[165]
PLA/Silk	Injection Molding	↑ Mechanical	-	Physiological environment	[166]
PLA/PBAT/silk	Injection Molding	↑ Mechanical	-	-	[167]
PLA/PCL/NP	Compression Molding	↑↑ Mechanical, thermal	-	Hydrolytic degradation	[168]
PLA/PCL/SF	Compression Molding	↑ Mechanical, thermal	-	Hydrolytic degradation	[168]
silk/PBS	Compression Molding	↑ Mechanical, thermal	-	-	[169]
wsilk/PBS	Compression Molding	↑ Mechanical, thermal	-	-	[170]
PHA/OSP	Compression Molding	↑ Mechanical, sustainability	Packaging, utensils	Soil degradation	[81]
MB/EE	3D Printing	↑ Mechanical, sustainability	Packaging	-	[161]

↑ Increasing properties; ↑↑ Major increasing properties if compared with other formulation of the same work.

## Data Availability

No new data were created or analyzed in this study. Data sharing is not applicable to this article.

## Abbreviations

PLA	Polylactic Acid
PCL	Polycaprolactone
PBS	Poly(butylene succinate)
PHAs	Polyhydroxyalkanoates
PHB	Polyhydroxybutyrate
PHBV	Poly(3-hydroxybutyrate-co-3-hydroxyvalerate)
PBAT	Poly(butylene adipate-co-terephthalate)
TPS	Thermoplastic Starch
PVA	Polyvinyl Alcohol
CA	Cellulose Acetate
AP	Artichoke Plant
BF	Bamboo Fiber
CBS	Cocoa Bean Shell
CBSW	Cocoa Bean Shell Waste
CH	Cocoa Husk
GP	Grape Pomace
HSF	Hazelnut Shell Flour
HS	Hazelnut Shells
HC	Hedysarum Coronarium
MS	Mango Seed
OW	Olive Wood
OFI	Opuntia ficus indica
OPP	Orange Peel Powder
RH	Rice Husk
MNRH	Micro–Nano Rice Husk
RS	Rice Straw
RSP	Rice Straw Powder
SSHP	Sesame Husk Powder
TPF	Tangerine Peel Flour
THP	Tomato Peel
WM/WW	Wheat Middling/Wheat Waste
PL	Pineapple Leaf/Pineapple Leaf Fiber
PO/POL/POS	Posidonia oceanica Leaves/Powder
AV	Aloe Vera
AB	Algal Biomass
BS	Babassu Shell or Residue
MO	Moringa oleifera
WE/ESP/WES	Waste Eggshell
FG	Fish Gelatin
PUS	P. undulata Shell
CSP	Crab Shell Powder
EE	Engraulis Encrasicolus (Anchovy Bones)
OSP	Oyster Shell Powder
WWF/WWP	Waste Wool Fiber/Waste Wool Powder
CNC	Cellulose Nanocrystals
MFC	Micro-Fibrillated Cellulose
NCs	Nanocellulose (agro-industrial)
WP	Waste Paper
MCC	Microcrystalline Cellulose
MB	Mater-Bi^®^
Inz	Inzea^®^
MC	Melt Compounding
IM/Inject. mold.	Injection Molding
CM/Compress. mold.	Compression Molding
HP	Hot Process
SBS	Solution Blow Spinning
FDM	Fused Deposition Modeling
SC	Solution Casting/Solvent Casting
UC mill	Ultra-Centrifugal Milling
HS mill	High-Speed Milling
NaOH	Sodium Hydroxide Treatment
HCl	Hydrochloric Acid Treatment
DCP	Dicumyl Peroxide
UV	Ultraviolet Radiation
NPK	Nitrogen, Phosphorus, Potassium (fertilizer)
